# Progress in the application of nanoparticles for the treatment of fungal infections: A review

**DOI:** 10.1080/21501203.2023.2285764

**Published:** 2023-12-08

**Authors:** Xinlin Zhu, Youming Chen, Dan Yu, Wenjie Fang, Wanqing Liao, Weihua Pan

**Affiliations:** a Department of Dermatology, Shanghai Key Laboratory of Molecular Medical Mycology, Second Affiliated Hospital of Naval Medical University, Shanghai, China; b Department of Infectious Diseases and Immunology, Shanghai Public Health Clinical Center, Fudan University, Shanghai, China; c Department of General Practice, Second Affiliated Hospital of Naval Medical University, Shanghai, China

**Keywords:** Fungal infection, antifungal, nanoparticle

## Abstract

The burden of fungal infections on human health is increasing worldwide. *Aspergillus*, *Candida*, and *Cryptococcus* are the top three human pathogenic fungi that are responsible for over 90% of infection-related deaths. Moreover, effective antifungal therapeutics are lacking, primarily due to host toxicity, pathogen resistance, and immunodeficiency. In recent years, nanomaterials have proved not only to be more efficient antifungal therapeutic agents but also to overcome resistance against fungal medication. This review will examine the limitations of standard antifungal therapy as well as focus on the development of nanomaterials.

## 1. Introduction

Fungi are complex eukaryotes with distinct morphological traits that thrive in varied ecological environments. The most clinically serious fungal infections occur in patients with low immune function or critical patients, such as patients with malignant cancer, acquired immunodeficiency syndrome (AIDS), large surgical wounds, and recipients of organ transplantation or haemodialysis (Zheng et al. 2018; Xiao et al. 2019; Kazakou et al. 2020; Wong et al. 2020).

The primary pathogens of invasive mycosis are *Candida albicans*, *Aspergillus fumigatus*, and *Cryptococcus neoformans* (Limper et al. 2017). Among them, *C*. *albicans* has the highest infection rate in intensive care units, whereas *A*. *fumigatus* is more common in patients with high-risk blood tumours and patients with the chronic obstructive pulmonary disease treated with glucocorticoids (Roca-Barcelo et al. 2020). *C*. *neoformans* primarily occurs in human immunodeficiency virus (HIV) patients, causing cryptococcal meningitis (Limper et al. 2017). Secondary fungal infections of the skin and mucous membrane as well as invasive infections aggravate the primary disease, damaging organ function and seriously affecting the prognosis. Although various new antifungal drugs have achieved certain therapeutic effects since the 1950s, the clinical fungal infection rate is continuing to rise (Stott et al. 2021). A possible explanation could be the mechanism of multiple drug resistance of fungi to existing antifungal drugs. Moreover, defects in the actual therapeutic efficiency, side effects, and activity of existing antifungal drugs hinder further clinical application.

In recent years, the feasibility and safety of nanoparticles (NPs) as antifungal drug delivery methods have been widely explored (Li et al. 2021). This paper will review the treatment of fungal infectious diseases and advancements in NPs across three distinct sections: (1) fungal infectious diseases, (2) existing treatment schemes and their limitations, and (3) characteristics and functionalization of antifungal NPs and their existing clinical applications.

## 2. Fungal infection

Fungi are an important class of microorganisms, which have a significant impact on human life. It is common for people to come in contact with and get infected with fungi through respiratory, cutaneous, and gastrointestinal routes (Chao and Vazquez 2021). Furthermore, fungal infections are more common among immunocompromised patients with HIV/AIDS (Limper et al. 2017), uncontrollable hyperglycaemia (Goswami et al. 2000), haematological diseases (Danek et al. 2020), and patients undergoing chemotherapy (Charpak-Amikam et al. 2022).

### 2.1. Candidiasis

*Candida* spp., the most usual pathogeny causing invasive mycotic disease, is the primary cause of all healthcare-associated bloodstream infections in the United States. And despite the use of antifungal therapy, the crude mortality rate of *Candida* spp. reached 40% (Wisplinghoff et al. 2004; Pfaller and Diekema 2007; Pfaller et al. 2019). Invasive candidiasis includes deep-seated infections and bloodstream infections. Specifically, invasive candidiasis contains intra-abdominal abscess, osteomyelitis (bone infection) and peritonitis with or without candidemia (Pappas et al. 2018). *Candida* spp. can cause vulvovaginal candidiasis or thrush, mucocutaneous candidiasis (Akpan and Morgan 2002; Cooke et al. 2022). According to the age and site of infection of patients, several distinguished clinical mucocutaneous candidiasis could be distinguished, such as erythema mycoticum infantile, *Candida* intertrigo, erosio interdigitalis blastomycetica, onychia, candidal paronychia, *Candida* onychomycosis, and oral candidiasis (Katoh 2009). These infections can frequently occur in patients with extremely compromised immunity and who have undergone invasive clinical procedures or catastrophic trauma (Pfaller and Diekema 2007; Brown et al. 2012). Among *Candida* species other than *C. albicans*, *C. glabrata* stands out as the primary culprit behind invasive candidiasis, with a consistently increasing number of reported cases in recent years (Pfaller et al. 2019). In recent times, the advent of *C*. *auris* has generated tremendous concern worldwide, and this fungus is regarded as the “superbug” for its high rates of transmission in clinical settings and multidrug-resistant properties (Lee et al. 2021).

### 2.2. Cryptococcosis

Cryptococcosis is an invasive fungal illness, primarily caused by *C*. *neoformans* and *C*. *gattii* (Buchanan and Murphy 1998; Ingavale et al. 2008). Although *C*. *neoformans* is responsible for 95% of human cryptococcal illnesses, *C*. *gattii* is gaining recognition as a worldwide pathogen (Huang et al. 2022). Cryptococcal meningitis is usually linked to advanced HIV infection and is responsible for 250,000 cases and 180,000 deaths worldwide annually (Iyer et al. 2021).

### 2.3. Aspergillosis

*Aspergillus* spp. cause chronic and invasive infections of the lungs, although they can disseminate to other organs as well. These invasive diseases are most likely to affect patients with highly compromised immune systems, including those receiving chemotherapy (strongly immunosuppressive), undergoing transplantation, and administered high-dose corticosteroids for prolonged periods (Kontoyiannis et al. 2010). Aspergillosis comprises both non-invasive and invasive forms. Non-invasive forms of aspergillosis encompass conditions like allergic fungal rhinosinusitis and allergic bronchopulmonary aspergillosis, while the invasive form comprises invasive pulmonary aspergillosis (IPA) and chronic pulmonary aspergillosis (Barnes and Marr 2006). IPA has been associated with respiratory viral infections, such as influenza A, avian influenza H7N9, respiratory syncytial virus, influenza B and severe acute respiratory syndrome coronavirus 2 (Dewi et al. 2021; Machado et al. 2021), and is believed to be secondary to epithelial injury within the airways, allowing for invasion by colonising *Aspergillus* spp. (Cadena et al. 2021). The annual rates of aspergillosis have improved significantly, whereas the mortality trend across aspergillosis subgroups has remained stable.

### 2.4. Dermatophytosis

Dermatophytosis is a skin infection caused by dermatophytes, which are typically harmless and represent a common type of infection worldwide, affecting approximately 20% of the global population. These fungi infect the stratum corneum, along with other keratinised tissues like nails and hair, and they propagate by secreting enzymes to break down keratin for sustenance. Nevertheless, in immunocompromised individuals, rare and severe diseases could be led, including severe conditions encompassing extensive or invasive dermatophytosis (Rouzaud et al. 2015). These conditions are documented in individuals with compromised immune systems, stemming from either inherent factors (autosomal recessive CARD9 deficiency) or acquired causes (including solid organ transplantation, autoimmune diseases necessitating immunosuppressive therapies, and HIV infection) (Rouzaud et al. 2015). The clinical symptoms of the infection lack specificity, and it can lead to lymph node and organ involvement. Establishing a diagnosis necessitates both mycological and histological assessments, but a unanimous consensus on treatment remains absent. Systemic antifungal medications, such as terbinafine and azoles, have demonstrated effectiveness. Nonetheless, the long-term prognosis and management of treatment are contingent upon the location and scope of the infection as well as the specific underlying immunodeficiency.

### 2.5. Pneumocystis pneumonia

Patients with HIV/AIDS are increasingly susceptible to pneumocystis pneumonia, with over 400,000 cases reported each year (Brown et al. 2012). Many individuals go undetected or have late diagnoses, particularly in resource-constrained settings. Depending on the patient group, comorbidity, and establishment of early diagnosis, the mortality of pneumocystis pneumonia varies from 10% to 30% or higher (Thomas and Limper 2004, 2007).

### 2.6. Zygomycosis

Zygomycosis refers to a group of uncommon but frequently fatal mycoses caused by *Zygomycetes*, which is subdivided into two orders: mucorales and entomophthorales (Gonzalez et al. 2002). Although percutaneous routes and cutaneous are also common, *Zygomycetes* are transmitted principally through inhalation of spores from environmental sources. The occurrence of zygomycosis is more common in susceptible hosts, such as patients suffering from haematologic or oncologic diseases and diabetics, newborns at high risk, patients with burns and so on (Roden et al. 2005).

### 2.7. Endemic mycoses

Blastomycosis, coccidioidomycosis, emergomycosis, histoplasmosis, paracoccidioidomycosis, sporotrichosis, and talaromycosis are among the most common endemic mycoses. Historically, these diseases occurred in a limited geographical range and were considered to be the primary factors contributing to both the incidence rate and mortality in the case of HIV/AIDS, other immunosuppressive diseases or the use of immunosuppressants. In addition, it is possible for the pathogen to enter through the airways or directly through the skin of the host (Queiroz-Telles et al. 2017).

## 3. Conventional therapeutic drugs for fungal infection

### 3.1. Existing clinical antifungal drugs

Compared with antibiotics, the methods of antifungal drugs are quite restricted. In addition to their distinctive cell wall structure, fungi share numerous similarities with host cells, which make the discovery of novel medications challenging (Arastehfar et al. 2021).

Amphotericin B (AmB), a polyene antifungal agent with *in vitro* activity against a variety of fungal pathogens, is commonly used in the clinic because of its wide antifungal spectrum and low drug resistance rate and can be used to treat invasive and severe fungal diseases. The mechanism of action of AmB involves binding to the fungal cell membrane ergosterol and producing an aggregate that creates a transmembrane channel through which the cytoplasmic contents (vital ions, small metabolites) leak out, resulting in metabolic disruption and cell death (Brajtburg et al. 1990). AmB is cytotoxic against both fungal and mammalian cells but more selective towards fungal cells because of its higher affinity to fungal ergosterol than cholesterol. However, the interaction between AmB and cholesterol in vital organs, including kidney and heart, causes serious side effects, such as severe nephrotoxicity (Hamill 2013). Despite the introduction of newer antifungal agents for the treatment of systemic mycoses, AmB is considered to be the established standard treatment for a range of severe, invasive fungal infections. AmB is efficacious against specific fungal infections, including cryptococcal meningitis. The principal adverse effects associated with AmB encompass nephrotoxicity, hypokalemia, hypomagnesaemia, and bone marrow suppression, with renal dysfunction being the primary concern in terms of AmB treatment-related toxicity (Hamill 2013). AmB has two formulations: deoxycholate AmB (D-AmB) and liposomal AmB (L-AmB). L-AmB is preferred over conventional D-AmB because it causes fewer infusion-related symptoms. Following intravenous delivery, the liposomal carrier remains physically and chemically unchanged for extended durations, resulting in a prolonged presence of AmB in the central bloodstream. In addition, L-AmB has the unique ability to extravasate into the infection site and administer the medication directly, without causing nephrotoxicity or neurotoxicity, which are commonly associated with conventional AmB formulations such as D-AmB (Hay 1994). In preclinical research, L-AmB achieved greater peak plasma concentration (Cmax) and area under the plasma concentration-time curve values compared to equivalent doses of D-AmB. Moreover, data from several clinical studies demonstrate that compared with D-AmB, L-AmB can be administered at significantly elevated dosages, leading to greater plasma exposure, heightened drug distribution in the lungs and central nervous system, improved antifungal effectiveness, decreased kidney toxicity, and the absence of significant new adverse effects (Groll et al. 2019).

Azole antifungals inhibit the synthesis of ergosterol and these drugs are suitable for oral administration in the treatment of persistent fungal infections. Various azole antifungals have diverse impacts on fungal diseases. For instance, fluconazole has proven highly beneficial in the treatment of cryptococcal meningitis and coccidioidal meningitis. Isavuconazole is recognised for its efficacy in treating mucormycosis, while itraconazole has emerged as the preferred treatment for lymphocutaneous sporotrichosis, as well as mild to moderately severe instances of histoplasmosis, blastomycosis, and paracoccidioidomycosis. Voriconazole is regarded as the preferred treatment for aspergillosis in both immunocompetent and immunocompromised individuals.

Echinocandins, which include caspofungin, micafungin, and anidulafungin were introduced in the early 2000s (Cortés et al. 2019). Echinocandins are water-soluble lipopeptides that exhibit fungicidal activity by inhibiting the synthesis of β-1,3-D-glucan (an essential component of the fungal cell wall). Due to the distinctive mechanism of action they employ compared to other antifungal medications, they can only be used for intravenous injection. Echinocandins target the fungal cell wall, thereby making them attractive owing to their lack of cross-resistance with other drugs, and their target is fungal with no mammalian counterpart. Therefore, they have good safety in clinical applications. Echinocandins have activity against *C*. *albicans*, non-*C*. *albicans* spp., and *Aspergillus* with intrinsic resistance to azole (Kofla and Ruhnke 2011). They are active against not only the planktonic form of *Candida* but also the biofilm (Simitsopoulou et al. 2013; Zuo et al. 2021).

Synthesised for the first time in 1957, 5-fluorocytosine (5-FC) is a synthetic antimycotic compound. It doesn’t exhibit any inherent antifungal activity until it penetrates fungal cells via the cytosine osmotic enzyme and is transformed into 5-fluorouracil by cytosine deaminase. After phosphorylation, it integrates into fungal RNA to block protein synthesis and affects *Candida*, *Cryptococcus*, *Aspergillus*, and dematiaceous fungi, causing chromomycosis (Fang et al. 2020). 5-FC can also be transformed into fluorodeoxyuridine phosphate and inhibit thymine synthase, DNA synthesis and division, and the action of nanodrug delivery system in the treatment of antifungal infection (Bhattacharya et al. 2020). Because the likelihood of drug resistance is high when 5-FC is administered alone, it is almost always used with another antifungal, usually AmB. In addition, 5-FC has adverse effects, such as liver toxicity and bone marrow suppression (Bouz and Doležal 2021). Despite substantial advancements in existing antifungal therapies, the outcomes are still unsatisfactory, largely due to the limited array of clinically accessible antifungal classes and the emergence of resistance to current antifungal agents, common drug side effects and emerging opportunistic pathogens (hepatorenal toxicity), and challenges in the treatment of biofilm infections. In addition to the inherent and acquired drug resistance of antifungal drugs, almost all existing drugs exhibit drug resistance in some fungi.

### 3.2. Prevalence of drug resistance

For a positive patient outcome, fungal infections need effective antifungal treatment. Because only a few classes of antifungal medications exist, the development of resistance to single drug classes and increasing multidrug resistance have a substantial effect on therapy. Azole resistance is one of the biggest hurdles to clinical success in *Candida* and *Aspergillus* spp., followed by echinocandin- and multidrug-resistance in select *Candida* spp., particularly *C*. *glabrata*.

While *C*. *albicans* is the most frequently encountered species in invasive infections across various institutions, non-*Candida albicans* spp., including *C*. *krusei* and *C*. *glabrata*, have gained global prominence due to their elevated levels of antifungal resistance, especially against fluconazole (Van Leth and Schultsz 2023). Recently, there has been growing interest in *C. auris*, both in popular media and the medical literature (Ostrowsky et al. 2020). This relatively recent species was first identified in 2009 when it was isolated from the external ear canal of a patient in Japan (Satoh et al. 2009), and the first instances of invasive infections were documented in South Korea in 2011 (Ahmad and Alfouzan 2021). Currently, *C. auris* is established as an endemic pathogen in India and South Africa, where it accounts for 15% and 5% to 30% of reported Candidemia cases, respectively (Ahmad and Alfouzan 2021; Seagle et al. 2021). In the United States, over 90% of the isolates of *C*. *auris* are resistant to fluconazole, 30% to AmB, and 5% to echinocandin (Seagle et al. 2021).

The increasing prevalence of azole resistance in *Aspergillus* spp., notably *A*. *fumigatus*, has caused significant concern because this class of antifungals is frequently used to treat *Aspergillus* infections. Resistance to azole drugs in *Aspergillus* has developed in individuals who have been exposed to azoles for an extended period. The initial cases of azole resistance were documented in patients undergoing treatment with itraconazole (Wiederhold and Verweij 2020). Multiple studies, particularly in Europe, have documented increased levels of azole resistance in *A. fumigatus*. In certain instances, these rates have escalated to as much as 28% in specialised healthcare facilities catering to patients with chronic pulmonary aspergillosis who have received long-term azole treatment (Bueid et al. 2010).

### 3.3. Mechanisms of drug resistance

Drug resistance is induced by molecular mechanisms that occur naturally in less vulnerable species and are acquired in vulnerable populations (Arastehfar et al. 2021). The mechanisms involved in drug resistance include alterations in drug-target interactions (Cortés et al. 2019), decreased cellular drug concentrations (Shapiro et al. 2011), and biofilm-related permeability barriers (Souza et al. 2022).

Extended exposure to azoles may clinically manifest as azole resistance in *A.* *fumigatus*, which can be attributed to point mutations occurring in CYP51A. This gene encodes the enzyme responsible for catalysing the final step of the ergosterol synthesis pathway (Seyedmousavi et al. 2014). Apart from probable clinical exposure, environmental contact with azole compounds utilised in agriculture and various other contexts can contribute to drug resistance (Verweij et al. 2007, 2009; Snelders et al. 2009). Instances of azole-resistant *A.* *fumigatus*-induced invasive aspergillosis have been documented among patients undergoing azole therapy, and this phenomenon is now becoming increasingly prevalent on a global scale (Meis et al. 2016; Resendiz Sharpe et al. 2018). Several azole-resistance pathways that do not involve *CYP51A* have recently been identified and involve upregulation of gain-of-function mutations in transcription factors (Liu et al. 2020), efflux pump genes (El-Ganiny et al. 2022), and point mutations in 3-hydroxy-3-methyl glutaryl coenzyme A (HMG-COA) reductases (Hmg1 and Hmg2) (Fraczek et al. 2013; Gonzalez-Jimenez et al. 2021).

5-FC functions as a prodrug that gains entry into cells via the cytosine permease Fcy2. Although 5-FC itself is not harmful, once it penetrates fungal cells, it undergoes conversion to the toxic compound 5-fluorouracil (5-FU) through the action of cytosine deaminase, an enzyme absent in human cells. In fungi like *Cryptococcus* and others, cytosine deaminase is encoded by the *FCY1* gene. Subsequently, 5-FU is metabolised by uracil phosphoribosyltransferase (*FUR1*), inhibiting both DNA and protein synthesis. Resistance mechanisms are well understood in other fungal pathogens, such as *C.* *albicans*, where resistance to 5-FC can be mediated through loss-of-function mutations in *FCY1*, *FCY2*, and *FUR1* (Billmyre et al. 2020). Nonetheless, in the cases of *Candida lusitaniae* and *Candida dubliniensis*, it is only mutations in *FUR1* that lead to cross-resistance to 5-FU. Conversely, in *Cryptococcus deuterogattii*, resistance to 5-FC is conferred by deletions in FCY2 (Bhattacharya et al. 2020).

Resistance to echinocandins is associated with repeated or ongoing medication and may develop immediately after a short period of treatment (Lewis et al. 2013). Echinocandin resistance is driven by changes in the activation of cellular stress and target enzyme (1–3)-D-glucan synthase (Cortés et al. 2019). Mutations occurring in the three extensively preserved hotspot regions of the FKS genes, which encode the catalytic subunit of (1–3)-β-D-glucan synthase, result in increased resistance of the target to echinocandins. Echinocandin-induced cell wall destruction triggers various stress regulatory pathways and compensatory mechanisms that result in the production of excess cell wall chitin, and paradoxically, fungal resistance adaptation to echinocandins (Walker et al. 2018). Lastly, the adaptability of fungus to sublethal dosages of antifungal drugs can lead to the establishment of resistance (Wiederhold et al. 2005).

## 4. Characteristics of NPs against fungal infection

Despite advances in targeted therapy against fungi, several treatment-related limitations, including low absorption of certain medications and the emergence of drug-resistant strains, have made the deployment of conventional fungal therapy a challenge (Kawasaki and Player 2005). Antifungal NP-based carriers could be utilised to address some limitations through targeted delivery and enhanced localisation, dosage reduction, better pharmacokinetics, and controllable drug release (Zhi et al. 2021). Moreover, NPs can be utilised to directly interact with a target fungus to generate an antifungal effect, for example, Ag NP-induced cellular toxicity (Feng et al. 2015) or function as a vehicle for additional compounds that can interact with the targeted fungus (Kischkel et al. 2020).

### 4.1. NP-based carriers increase efficacy, and concurrently, reduce drug-related side effects

The goal of nanotechnology is to use nanostructures as pharmaceuticals to improve the efficacy of current antifungal therapies and reduce their drug-related side effects. AmB is a common polyene known for its broad-spectrum antifungal properties, but its utility is restricted due to its adverse effects. In this regard, Saldanha et al. (2016) created a magnetic carrier nanocomplex consisting of magnetite nanoparticles coated with a double layer of lauric acid, onto which AmB was loaded on the surface. Compared with AmB alone, the NP-AmB complex not only alleviated pulmonary symptoms but also reduced systemic toxicity.

### 4.2. Unmodified NPs against fungal infections

Nanotechnology offers novel approaches that do not rely on inhibiting drug resistance pathways for treating fungal infections. Unmodified NPs alone showed satisfactory antifungal activity (Paraguay-Delgado et al. 2022). The underlying mechanism of these antifungal platforms is mediated by reactive oxygen species (ROS), permeability of the membrane, metal ions transport, pH gradient, and photo- and magnetic hyperthermia, where fungi are unable to survive (Weldick et al. 2022).

Studies on antibacterial activity have found that Ag NPs kill bacteria at a relatively low level, without acute cytotoxicity in humans (Gurunathan et al. 2014). Using *in vitro* experiments, Panácek et al. (2009) reported that Ag NPs have strong antifungal efficacy against pathogenic *Candida* spp. at 1 mg/L, which is comparable with conventional antifungal drugs.

NPs can kill fungal cells embedded in biofilms (Joshi et al. 2022). Jothiprakasam et al. (2017) tested the antifungal properties of zinc oxide (ZnO) NPs on fluconazole-resistant strains of *C*. *tropicalis in vitro* and reported that ZnO NPs presented satisfactory antifungal activities at 5.42 μg/mL.

### 4.3. Toxicity of nanoparticles

Although nanotechnology offers several antifungal treatment options, the negative effects of nanomaterial-based therapy must also be considered. The mechanisms involved in NP-mediated toxicity to fungi, for example, may be active in humans. Studies on the toxicity of Ag NPs have estimated the viability of Ag NPs and ROS generation in cell lines (Lesniak et al. 2005; Arora et al. 2008). Hsin et al. observed Ag NPs mediated mitochondria-dependent apoptosis in mouse fibroblasts through ROS generation and JNK activation (Hsin et al. 2008). According to Asharani et al. (2009) although Ag NPs are effective in treating microbial infections (including those caused by *C*. *albicans*), destruction of the mitochondrial respiratory chain and increase in ROS production destroy DNA structure. Another study evaluated the uptake and cytotoxicity of Ag NPs in human peripheral blood mononuclear cells (PBMCs) using polyvinylpyrrolidone-coated Ag NPs and AgNO_3_ controls and observed that compared with controls, Ag NPs were more toxic to PBMCs when normalised to the amount of cell-associated Ag (Pourhoseini et al. 2021). Although higher concentrations of Ag NPs are advised for therapeutic applications, it contradicts the obtained toxicity data (Rajan et al. 2022). Further studies aim at the systematic evaluation of toxicity in conjunction with appropriate controls, and detailed studies on the development of protein corona and the (bio)transformation of Ag NPs in specific biosystems (cell types) will contribute to a broader understanding of NP-induced toxicity.

## 5. Recent advances in NPs against fungal infection

### 5.1. Polymeric NPs

Polymeric NPs were developed to prevent pharmaceutical loss and early disintegration following chemical or enzymatic inactivation (Zielińska et al. 2020). Poly (lactic-co-glycolic acid) (PLGA) and Chitosan are the two major polymer NP-manufacturing materials (Park et al. 2010).

To improve NP adherence to *C*. *albicans* cell walls, PLGA NPs modified with glucosamine (PLGA-GlcN) were developed (Mohammadi et al. 2017). Nystatin-loaded PLGA-GlcN exhibited higher antifungal activity of nystatin against a *C*. *albicans* strain than PLGA NPs and pure nystatin (Kim et al. 2017). In another study, the preparation of chitosan NPs loaded with ketoconazole was more effective against *A*. *niger* than unfunctionalized NPs or ketoconazole alone (Kumar et al. 2016).

Ferulic acid (FA) and its derivatives inhibit biofilm formation in *C*. *albicans* (Canturk 2018). Nonetheless, the use of FA is restricted due to its poor permeability and stability *in vivo* (Canturk 2018). To overcome this problem, Panwar et al. (2016) developed FA-encapsulated chitosan NPs, which exhibited effective antibiofilm activity against *C*. *albicans in vitro*.

**Figure 1.**
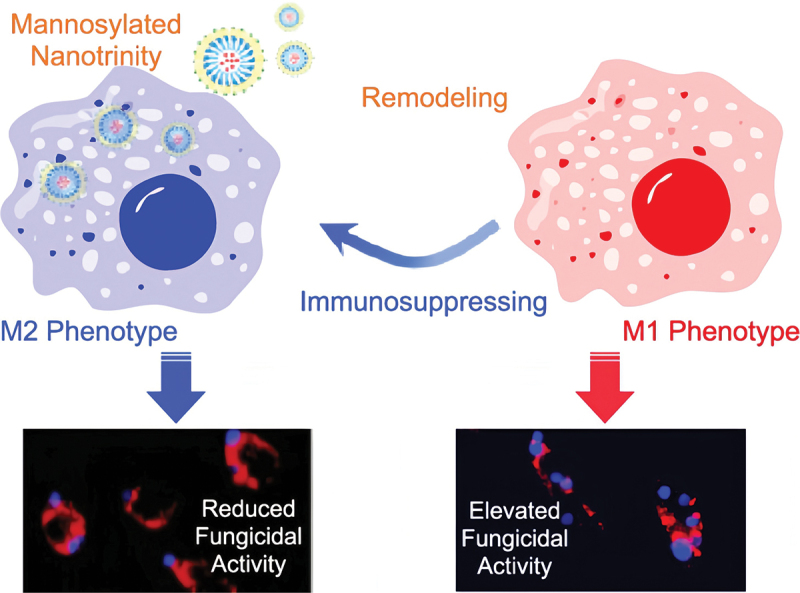
Mannosylated nanotrinity synthesis and macrophage remodelling. Mannose was covalently conjugated with chitosan oligosaccharides and then the product was incubated with imatinib-laden nanoparticles obtaining the chitosan-coated nanoparticles. The mannosylated nanotrinity developed in this study could significantly induce macrophage remodelling *in* *situ* by the two-pronged process, “turning on” M1 phenotype polarisation meanwhile “shutting off” M2 phenotype polarisation, and thus allowed to eradicate *C.* *albicans* infection (Gao et al. 2020).

Macrophages are the first line of defence, recognising and engulfing foreign agents, such as fungi (Seider et al. 2010). M1 macrophages play an important role in host defence against various diseases via phagocytosis and antigen presentation, whereas M2 macrophages are involved in the negative regulation of the immune system (Mosser 2003). Employing phagocyte-mediated immunotherapy to treat *C*. *albicans* infection is a promising approach (Bruno et al. 2020; Charpak-Amikam et al. 2022). Gao et al. (2020) used imatinib-laden NPs, coated with chitosan and linked with mannose, for macrophage M1 polarisation. The mannosylated nanotrinity produced could stimulate macrophage remodelling *in situ* through a two-pronged mechanism: “turning on” M1 phenotype polarisation and “turning off” M2 phenotype polarisation, thereby eradicating *C*. *albicans* infection (Gao et al. 2020) (Figure 1).

### 5.2. Mesoporous silica NPs

Mesoporous silica NPs (MSNs) with a unique mesoporous structure have been investigated as efficient drug delivery methods for a range of medications to combat different illnesses (Tang et al. 2012). Furthermore, MSNs have been shown to be ideal candidates for transporting various medicines or nucleic acids (DNA, siRNA) to improve treatment efficacy against multidrug resistant infections (Torella et al. 2010).

**Figure 2.**
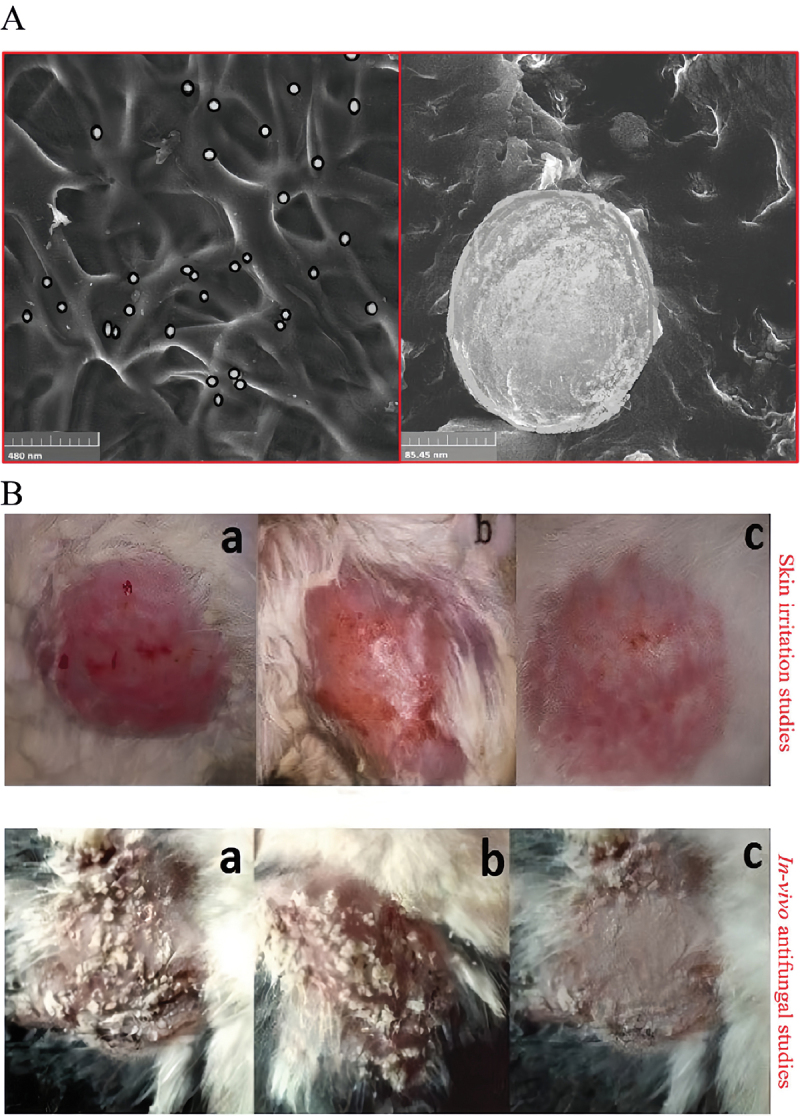
The functional assessment of the econazole-triamcinolone loaded silica nanoparticles. Maheen et al. mixed MSNs with econazole nitrate (EN) and triamcinolone acetonide (TA) to accelerate healing. In the *C.* *albicans*-infected animal models, the EN-TA-loaded MSNs exhibited superiority in anti-fungal activity and wound healing, compared with EN/TA alone. (A) SEM analysis of EN-TA-MSNs. (B) Skin irritation studies and *in* *vivo* antifungal studies in control group (a), group treated with pure EN-TA suspension (b), and group treated with EN-TA loaded MSNs (c) (Maheen et al. 2022).

Triamcinolone acetonide (TA) is used to treat various skin conditions. However, its use is restricted because of its low solubility in most pharmaceutical excipients, poor penetration, and high skin irritation (Agrawal et al. 2010). Maheen et al. (2022) mixed MSNs with TA and econazole nitrate (EN) to accelerate healing (Figure 2a). In *C*. *albicans*-infected animal models, MSNs loaded by EN-TA represented better antifungal activity and wound healing than EN/TA alone (Maheen et al. 2022) (Figure 2b).

Severe systemic infections, including sepsis, may occur when *C*. *albicans* enters the circulation and causes immunological dysregulation (Chen et al. 2017; Russell et al. 2019). The accumulation of activated macrophages and the development of hazardous microthrombi, produced due to infection-induced inflammatory storms, is an important consequence of systemic infections (Benjamin et al. 2018). During pathogen engagement, macrophages usually phagocytise the pathogens and rapidly activate NADPH oxidases, resulting in the production of ROS (Roca et al. 2019). Furthermore, the binding of macrophage receptors (e.g. Toll-like receptors) to pathogenic cells may trigger transcriptional cascades, resulting in fast synthesis of proinflammatory cytokines derived from the endoplasmic reticulum (ER), as well as ER stress for further cytokine production (Grover et al. 2018). The pathogen infection-responsive cap containing the ROS-cleavable boronobenzyl acid linker and bovine serum albumin and large-pore MSN grafted by an ER-targeting peptide constitutes a nanoplatform, which effectively suppressed proinflammatory cytokine secretion, pathogen growth, and ER stress. Moreover, it protected mice against mortality and organ failure in an *in vivo* systemic infection model (Zhao et al. 2022).

### 5.3. Lipid-based NPs

Lipid-based NPs have been extensively developed as antimicrobial carriers, primarily by using liposomes and solid lipid NPs (SLNs) (Cowen et al. 2014). The use of lipid-based NPs as an alternative for delivering the therapeutic component to the target location has broadened the therapeutic profile and reduced fungicide-associated adverse events (Cowen et al. 2014).

#### 5.3.1. Liposomes

Liposomes are spherical synthetic vesicles formed by a lipid bilayer that is typically composed of phospholipids (Akbarzadeh et al. 2013). Liposomes stand as one of the most widely employed materials for drug delivery, due to their intrinsic biocompatibility, capacity to encapsulate both hydrophobic and hydrophilic and minimal toxicity medicines (Jarvis et al. 2022). Liposomal drug delivery methods have been utilised to successfully treat various microbial infectious diseases compared with traditional dosage forms (Abu Lila and Ishida 2017; Nisini et al. 2018).

AmB can bind cholesterol and produce toxicity in mammalian cells, particularly in cholesterol-rich kidney cells (Saxena et al. 1998). Owing to the nephrotoxicity of standard AmB, an alternative delivery strategy using liposomes (Ambisome®; L-AmB) was developed (Mondal et al. 2014).

#### 5.3.2. Solid lipid NPs (SLNs)

In the 1990s, Solid lipid NPs (SLNs) were developed as a feasible drug carrier alternative to polymeric NPs, emulsions, and liposomes (Khames et al. 2019). SLNs are an intriguing alternative for medicine delivery because of the lack of organic solvents (Sandhu et al. 2021). Most SLNs are spherical particles, with diameters ranging from 100 to 1,000 nm. Medication molecules enclosed in SLNs are released continuously because saturated lipids break down slowly. Thus, the composition of lipid excipients has a significant impact on the biological destiny of drug molecules buried in the solid crystalline matrix as well as SLNs (Mu and Holm 2018). Furthermore, because SLNs have small size and lipid solubility, they can penetrate biological barriers, such as the blood brain barrier (Khan et al. 2018) and exhibit lower absorption by the reticuloendothelial system (Kakkar et al. 2011). Extensive research has been conducted on antifungal solid lipid nanoparticles (SLNs) in the context of drug-resistant *Candida* infections (Vaghasiya et al. 2013). Moazeni et al. synthesised fluconazole-loaded SLNs and tested the effectiveness of the best formulation against fluconazole-resistant *Candida* strains (Moazeni et al. 2016).

#### 5.3.3. Nanostructured lipid carriers

Nanostructured lipid carriers (NLCs) are biodegradable and biocompatible particles, blending with solid lipids and a proportion of liquid lipids obtained from natural origins (Khan et al. 2015). As second-generation carriers, NLCs can address the drawbacks of SLNs (Soliman 2017). NLCs have superior features that allow long-term stability and greater lipophilic drug retention capacity because of the liquid lipid fraction, making this type of device better for drug delivery (Varela-Fernández et al. 2022). In several studies, itraconazole integrated into NLCs demonstrated over 98% encapsulation efficiency and maintained stability even after a storage period of 6 months (Pardeike et al. 2016; El-Sheridy et al. 2019). In addition, researchers detected radiolabeled NLCs in rat blood for up to 24 h following intravenous administration (Beloqui et al. 2013).

### 5.4. Noble metal NPs

Surface stability is one of the most essential features of noble metal NPs in the biomedical area. This feature allows NPs to be surface modified with biological and organic compounds and polymers. Biomolecule-coated noble metal NPs have unique characteristics that would be difficult or impossible to replicate with synthetic materials (Rosi and Mirkin 2005), such as delivering biomacromolecules efficiently while minimising cytotoxicity (Lytton-Jean et al. 2011). Many metal-based drug delivery systems, such as silver and magnetic NPs, have been used to increase AmB distribution and achieve synergistic antifungal actions (Paulo et al. 2010; González et al. 2015). Kischkel and their team investigated the efficacy of Ag NPs in conjunction with propolis extract in combatting mature biofilms of *Candida* species and various other fungi. These findings revealed that the concentration required for the formulation’s antifungal activity was lower than the concentration that exhibited cytotoxic effects (Kischkel et al. 2020).

## 6. Conclusions and prospect

Over the past few decades, nanomedicine has assumed a pivotal role within the global healthcare sector. NPs are solid colloidal particles characterised by at least one dimension falling within the 1 to 100 nm range, with most materials employed in drug delivery falling between 100 and 200 nm. NPs exhibit exceptional capabilities, including minimal toxicity, chemical stability, surface functionalization, and biocompatibility. These advantages render them extensively applicable in various biomedical contexts. Given that antifungal agents could engender undesirable side effects such as diarrhoea, elevated body temperature, and heightened risk of renal failure, the quest for alternative treatment modalities to combat fungal diseases becomes imperative. Nanotechnology is one of the promising technologies for developing novel antifungal therapies due to its capacity for delivering drugs with precision, it enables the reduction of toxicity and treatment expenses.

NPs synthesis methods can be categorised into three types: chemical, physical, and biomediated (Koul et al. 2021). The biosynthesis approach has various merits compared with the physical approach (temperature, pressure, and energy) and the chemical approach (sol-gel, chemical etching, atomic condensation, spray-mediated pyrolysis and laser pyrolysis) because it is less toxic and more environmentally friendly (Koul et al. 2021; Karunakaran et al. 2023). This approach employs a range of stabilising and reducing agents, including plants, microorganisms, and certain natural compounds, for the synthesis of NPs. A study reported that Pt NPs, a type of organic NPs, demonstrated antifungal efficacy against multiple fungal strains, including *P*. *drechsleri, C*. *acutatum*, *C*. *fulvum*, *D*. *bryoniae*, and *P*. *capsici*. Research has indicated that Pt NPs, when combined in a nanomixture, enhance antifungal attributes by inducing membrane rupture, elevating ROS levels, altering mycelial structure, and leading to DNA impairment and cellular disintegration. The mechanism of fungicidal activity exhibited by biosynthesised metallic nanoparticles proves to be more efficient in comparison to traditional antibiotics such as amphotericin and fluconazole. Moreover, Ag NPs obtained from plant extracts demonstrated enhanced membrane disruption in Candida species when compared to organic NPs. This disruption was achieved by interfering with the fungus’s intracellular components and inducing cellular damage. NPs obtained via nonbiological approaches, such as physical and chemical methods can be toxic for patients who are severely ill or severely immunocompromised. However, biomediated NPs have fewer toxic effects and can be used to treat various diseases. Nanomedicine encompasses the application of nanoparticles for both therapeutic and diagnostic objectives and has been used in many therapeutic fields, particularly cancer, where the use of nanomedicine has greatly improved the safety and efficacy of common anticancer drugs. Indeed, NPs are as important for treating fungal and bacterial infections as they are for treating tumours. Nanotechnology makes it possible to develop formulations that can improve both treatment effectiveness and the quality of life for patients by mitigating side effects, especially during extended therapy. Although the use of NPs for fungal infectious diseases is in its early stages, preliminary research shows that this method has excellent prospects. Nanotechnology is developing rapidly in China. Thus, developing nanotechnology, particularly biomediated NPs in the treatment of fungal infections, maybe a good direction for us in the future.
